# High efficacy full allelic CRISPR/Cas9 gene editing in tetraploid potato

**DOI:** 10.1038/s41598-019-54126-w

**Published:** 2019-11-27

**Authors:** Ida Elisabeth Johansen, Ying Liu, Bodil Jørgensen, Eric Paul Bennett, Erik Andreasson, Kåre L. Nielsen, Andreas Blennow, Bent Larsen Petersen

**Affiliations:** 1 Department of Plant and Environmental Sciences, University of Copenhagen, DK-1871 Frederiksberg C, Denmark; 2 Copenhagen Center for Glycomics, Departments of Cellular and Molecular Medicine and Odontology, Faculty of Health Sciences, University of Copenhagen, DK-2200 Copenhagen N, Denmark; 3 Resistance Biology, Plant Protection Biology, Swedish University of Agricultural Sciences, Alnarp, Sweden; 4 Department of Chemistry and Bioscience, Aalborg University, Aalborg, Denmark

**Keywords:** Molecular engineering in plants, Plant breeding

## Abstract

CRISPR/Cas9 editing efficacies in tetraploid potato were highly improved through the use of endogenous potato *U6* promoters. Highly increased editing efficiencies in the Granular Bound Starch Synthase gene at the protoplast level were obtained by replacement of the Arabidopsis *U6* promotor, driving expression of the CRISPR component, with endogenous potato *U6* promotors. This translated at the ex-plant level into 35% full allelic gene editing. Indel Detection Amplicon Analysis was established as an efficient tool for fast assessment of gene editing in complex genomes, such as potato. Together, this warrants significant reduction of laborious cell culturing, ex-plant regeneration and screening procedures of plants with high complexity genomes.

## Introduction

Genome editing provides an efficient route of translating genetic knowledge into improved crop varieties in the field. In contrast to many breeding techniques, CRISPR-Cas genome editing minimizes introduction of undesired mutations^1^, which requires backcrossing and/or extensive selection to identify vigorous offspring. This becomes important in outbreeding crops and especially in those with complex genomes, where genome editing enables breeding of desired traits in existing elite cultivars, without compromising existing good agronomic performance^2^. Elite cultivars of tetraploid potato, *Solanum tuberosum*, are known to be extremely genetically diverse with a very high Small Nucleotide Polymorphism (SNP) frequency^3,4^; and are thus propagated clonally to maintain agronomic traits that are the result of balancing such overwhelming genetic diversity. Many traits can be improved by loss of function alleles, for example generated through CRISPR/Cas9 induction of the Non Homologous End Joining (NHEJ) pathway^5^. The most famous example is mlo-based resistance to powdery mildew^6,7^.

High efficient CRISPR/Cas9 editing are in many plants complicated by the presence of complex and high ploidy genomes and inefficient or poorly controlled delivery of the CRISPR/Cas9 components to cells with regenerative potential. Fluorescence Activated Cell Sorting (FACS) of cells expressing GFP tagged CRISPR/Cas9 is regularly used for enrichment of edited cell populations in mammalian cell systems^8^ and more recently also expanded to plant protoplast cells^9^. The Indel Amplicon Analysis (IDAA)^10^ technique allows for fast and direct assessment of insertions/deletions (indels), with a sensitivity down to + /−1 bp, without the need for in depth Sanger sequencing^11^. IDAA was recently used for editing scoring of protoplast cell populations^9^, and in the present study the use of IDAA was expanded and adapted to plants with complex genomes, such as potato, where it proved an efficient and fast tool for editing assessment.

Genome editing has been applied to several gene targets in potato albeit with moderate editing frequencies in protoplasts and subsequent regenerated ex-plant shoots. In one study TALENs were targeted to the acetolactate synthase gene resulting in 7–8%, 11–13%, 10% editing at the protoplast, calli, and regenerated shoot/ex-plant levels, respectively; but full allelic edited ex-plants were not obtained^12^. In another study an vacuolar invertase gene was targeted by CRISPR/Cas9. Here 18 of 600 shoots displayed editing and 5 (0.8%) had editing in all four alleles^13^. A recent study where the potato granule bound starch synthase (GBSS) gene was targeted by CRISPR/Cas9, showed that replacement of the standard *Arabidopsis thaliana U6*-*1* (*AtU*6-1) promoter, driving expression of the guide RNA, with a endogenous potato *U6* promoter resulted in a doubling in editing frequency from ca. 5% to 10% in regenerated ex-plant lines of which 2% displayed full allelic editing^14^. The presence of a single wild-type allele was sufficient for conferring significant amounts of the GBSS gene product amylose thus demonstrating that KO of all four alleles is necessary for producing amylopectin (waxy) potato starch devoid of amylose^14^. Similarly, the endogenous cotton (*Gossypium hirsutum*) *GhU6.3* promoter also resulted in increased CRISPR/Cas9 editing as compared to the *AtU6*-*29* promoter in a transient reporter expression system in cotton^15^.

In this study, we significantly improved CRISPR/Cas9 editing efficacy by applying endogenous potato *StU6* promoters for driving the CRISPR component of the CRISPR/Cas system, and demonstrate that this optimization has a dramatic effect on editing frequencies at both the protoplast and shoot/ex-plant level.

## Results

### Retrieval of endogenous U6 promotors and editing analysis at the protoplast level

**Figure 1:**
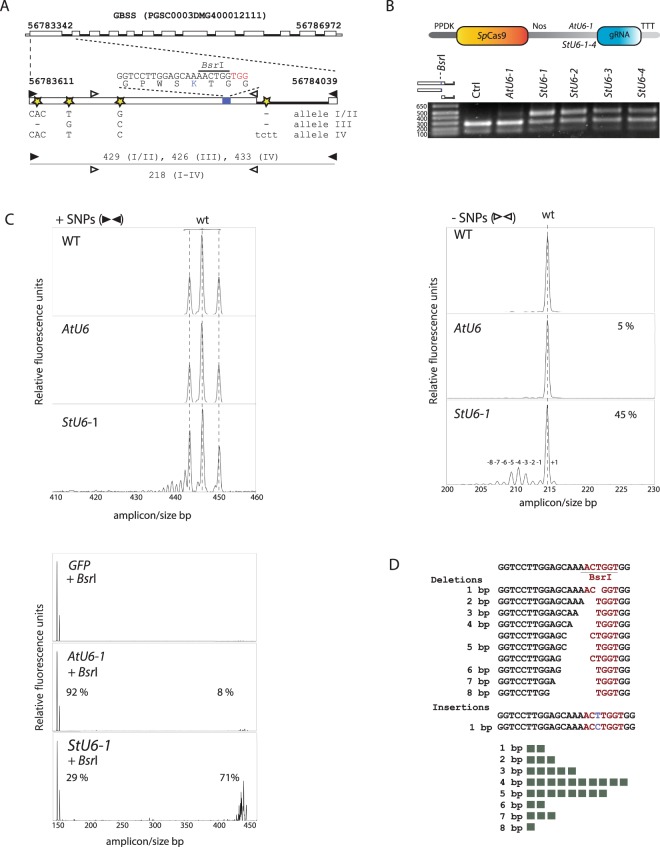
U6 promoter efficacy analysis at the protoplast level. *StU6* promoters were here defined as 350 nt upstream of the transcription start site (Supplementary Fig. 1 and Supplementary Fig. 2D), and the 257 bp *AtU6-1* promoter of *Arabidopsis thaliana* was obtained from the vector pHBT-pcoCas9^27^. (**A**) Structure of the Granular Bound Starch Synthase (GBSS) gene^3^ with the guide RNA (gRNA1) targeting part of the conserved motif and proposed active site KTGGL^23^. gRNA1 includes a diagnostic *Bsr*I restriction enzyme site spanning the *Sp*Cas9 cleavage site^28^, −3 bp upstream of the Photospacer Adjacent Motif (PAM) (red), which upon digestion yields the allele specific fragments: allele III (286, 140), allele I and II (289, 140) and allele IV (289, 144). The outer most primer set includes the SNPs (+SNPs) and gives rise to PCR amplicons of 426 (allele III), 429 (alleles I and II) and 433 bp (allele IV), and the innermost (-SNPs) to a PCR amplicon of 218 bp (all alleles). These length SNPs were conserved between the cultivars Desirée and Wotan. (**B**) Construct design and *StU6-1-4* versus *AtU6-1* promoter analysis at the cell pool (protoplast) level as evidenced by the presence of indel mediated destruction of the *Bsr*I site (*Bsr*I resistant band). (**C**) Indel Detection by Amplicon Analysis (IDAA) chromatograms for the + SNP and –SNP PCR amplicons of WT and *AtU6-1* and *StU6-1* derived indels. + SNP IDAA reveals allele complexity, while –SNP and *Bsr*I digested + SNP amplicons permit estimation of editing efficacy. (**D**) Sequence analysis of 34 individual clones of the *Bsr*I resistant band of *StU6-1* (**B**) isolated and cloned into the pJet vector, confirmed the indel distribution of the IDAA (Fig. 1C, panel: *StU6-1*, -SNP) also peaking at −4 bp deletions. WT peak positions are indicated by dotted lines. PPDK and NOS designate Pyruvate phosphate dikinase promoter and nopaline synthase terminator, respectively. All experiments were done in Desirée, except for panel C (upper right side) and D, which were done in the cultivar Wotan.

To identify and retrieve *StU6* promoters, we used sequences described in^16–18^ to search the potato genome. Upstream 5′ flanking sequences of the *StU6* promoters were retrieved and used to PCR amplify the promoter regions from the cultivars Desirée and Wotan. Four promoter sequences were identified of which two were hitherto unannotated (*StU6*-2 and *StU6*-3) and one (*StU6*-*4*) displayed size polymorphism (*StU6*-*4a* and *StU6*-*4b*). An alignment of the retrieved *U6* promoter sequences displays an overall high heterogeneity with respect to length and composition (Supplementary Fig. 1). The four potato *StU6*-*1*-*4* promoters, defined as 350 bp upstream of the *U6* gene start, were cloned to replace the *Arabidopsis thaliana AtU6*-1 promoter, thus driving expression of the target GBSS gRNA1 and gRNA2 in constructs also expressing the *Sp*Cas9 enzyme (Fig. 1A, Supplementary Fig. 2). In our initial designs we selected gRNAs targeting exon 1 of GBSS with diagnostic restriction sites for scoring editing. Potato leaf-derived protoplasts were isolated and the CRISPR/Cas9 expressing constructs (Supplementary Fig. 2) were delivered by polyethylene glycol (PEG) transformation as earlier described^12^. Indels were initially scored by PCR amplification of the targeted region from pools of protoplasts harvested 24 hrs after transformation followed by restriction enzyme digestion. This revealed a significant increase in editing for the endogenous *StU6-1-4* promoters when compared to the *AtU6*-*1* promoter as judged by restriction enzyme resistant band intensities (Fig. 1B). The experiment was repeated three times with overall similar results (data not shown). High resolution assessment of indel formation and distribution at the cell pool level was carried out through the use of the Indel Amplicon Analysis (IDAA) technique (Fig. 1C, Supplementary Fig. 3) and analyzed in detail by sequencing (Fig. 1D). When applied on the cultivar Wotan similar increases in editing were observed albeit at lower absolute editing frequencies (Supplementary Fig. 3). We speculate that subtle differences in chromatin structure, such as methylation status or packaging, or other factors between the two cultivars may explain these differences.

We expanded IDAA by restriction enzyme digestion of the IDAA samples, thus separating WT and indel peaks and allowing for direct assessment and quantification of only the indel derived peaks. Peak quantification revealed complete removal of the WT peaks in the Ctrl sample and a ca. 9 fold increase, from 8% to 71%, of edited alleles upon replacement of the *AtU6*-1 promoter with the *StU6*-*1* (Fig. 1C) or *StU6*-*2* (data not shown) promoter in protoplasts of the cultivar Desirée. The restriction enzyme resistant band of the *StU6*-*1* experiment (Fig. 1B) was cloned, sequenced and the indels scored, showing a distribution of deletions peaking at −4 bp, a few 1 nt additions (Fig. 1D) as well as large insertions (data not shown). IDAA and sequence analysis were consistent. The same analysis was applied on a second target in exon 1 of GBSS, gRNA2, which also showed higher efficacy using the *StU6* promoters compared to the *AtU6*-1 promoter, albeit with GBSS-gRNA2 having lower general editing efficacy (Supplementary Fig. 4). The indel distribution of these two gRNA target sites, i.e. the high prevalence of −4 and −1 deletions, respectively, is in agreement with a recent study, where Cas9 editing of a vast number of gRNAs/targets showed that each gRNA conferred individual cell-line-dependent bias toward particular Cas9 editing outcomes^19^. When exon 1 of GBSS was subjected to five selected *in silico* gRNA prediction servers^19^, the highly *in vivo* efficient GBSS-gRNA1 (Fig. 1) was ranked highest by the efficiency score in three of the predictors, but with a specificity score ranking it as #23 or #24 of 36 possible gRNA’s in the two predictors that allowed off-target assessment  (Supplementary Fig. 5). The non-trivial weighting of gRNA efficiency and specificity ranking when selecting gRNA targets may be augmented by unreliable or inadequate genome sequence information. While traditional breeding techniques that use mutagenic chemicals or irradiation result in mutation frequencies of 0.2–5 × 10^–3^^20^, only four off-target mutations could be attributed to CRISPR/Cas9 activity in tetraploid cotton analysed by whole genome sequencing^1^. This is less than 1% of the 466 SNPs and 77 indels identified as spontaneous mutations and in agreement with the recently reported none or very low off-target mutation frequencies in mammalian cells^21^.

### Ex-plant molecular and phenotypic characterization

**Figure 2:**
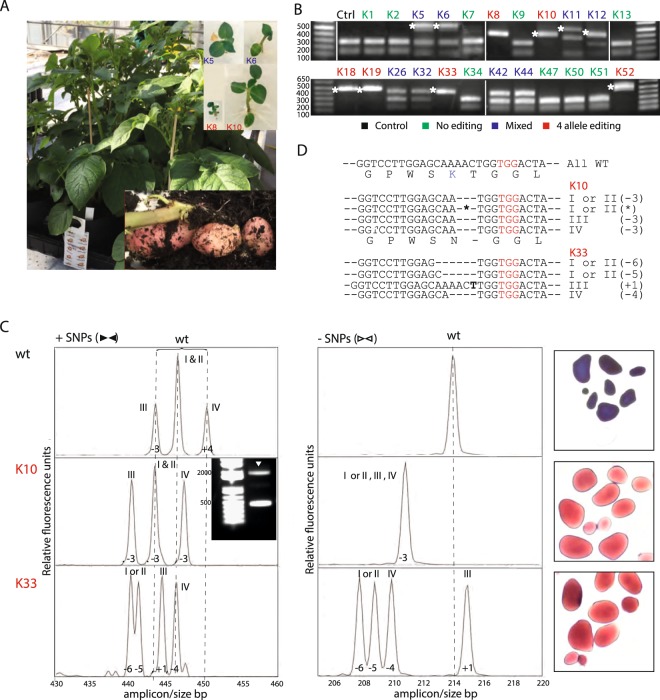
Amylopectin only potato: Geno- and phenotypic analysis of GBSS loss of function ex-plants. (**A**) Regenerated ex-plants from tissue culture with resulting potato tubers.(**B**) In total, 23 randomly selected shoots/ex-plants were screened to identify indels using the *Bsr*I restriction enzyme digestion. K8, K10, K18, K19, K33 & K52 appeared to have editing in all four alleles (fully *Bsr*I resistant), K11, K12, K26, K32 & K42 displayed a mixture of WT type and edited alleles, while K1, K2, K7, K9, K13, K34, K47, K50 & K51 appeared to be un-edited. * denotes insertions. K5 and K6 appear at a first glance to be mixed, but sequence analysis revealed that the *Bsr*I digestion bands were derived from plasmid insertions comprising the gRNA1 sequence, which reintroduced the *Bsr*I site and that K5 and K6 in fact had full allelic editing. (**C**) WT and the full allelic edited ex-plants, K10 and K33, were corroborated by IDAA of the larger (+SNP) region and the inner smaller (-SNP) region without length SNPs (Fig. 1C). WT peak positions are indicated by dotted lines. Lugol staining was used to analyse tuber starch from greenhouse grown plants (Supplementary Fig. 6). The presence of amylose gives rise to the dark blue colour (WT) tubers, while amylose free/’amylopectin only’ yields the red-brownish color. Additional shoots/ex-plants selected for IDAA analysis of which a subset was propagated to set tubers were also stained with lugol^29^ (Supplementary Fig. 7). (**D**) Sequence analysis confirmed that both K10 and K33 were full allelic edited. K10 had a 3 bp deletion in allele III, IV and in one of the alleles I or II and a 990 bp insertion (denoted ‘*’ left to the sequence, see also arrow on gel insertion (Fig. 2C)) in the other as also evident in the IDAA analysis displaying 1:1:1 ratio of the three chromatographic peaks. K33 had 6 bp and 5 bp deletions in allele I and II, a 4 bp deletion in allele IV and a 1 bp insertion in allele III.

Protoplast isolation, transformation, alginate embedment, shoot and ex-plant generation and transfer to soil for phenotypic scoring of starch composition in the tubers is outlined in Supplementary Fig. 6. Twenty three shoots/ex-plants were randomly selected and analyzed and out of these eight (35%) displayed full allelic editing, six appeared to have editing in 1–3 alleles, and nine were un-edited as evidenced by restriction enzyme analysis. Eleven out of the 23 shoots/ex-plants had plasmid derived insertions (Fig. 2B, left panel) and one displayed a dwarfed phenotype (Fig. 2B, right panel, plantlet K8). The high frequency of insertion mutants is in agreement with a recent study in potato, where CRISPR/Cas nucleoproteins were assembled *in vitro*. Here, an unexpected high frequency of insertions of both potato genomic DNA and fragments derived from the plasmid used for *in vitro* transcription of the gRNA was observed^22^. Molecular and phenotypic analyses of shoots/ex-plants initially selected for further analysis are outlined in Supplementary Fig. 7.

It was previously shown that full allelic knock-out of the GBSS gene is necessary for obtaining amylose-free starch^14^. Interestingly, two of the plants with four allele editing and amylose free starch, K10 and K33 (Fig. 2C), displayed three or six nucleotide in frame deletions in three or one allele, respectively (Fig. 2C,D), which resulted in elimination of the K-codon of the proposed ADPG binding site in the KTGGL motif^23^. The lack of amylose in K10 and K33 thus confirmed the importance of this amino acid for GBSS activity.

Noteworthy, our initial sequencing based characterization of the target region of the single copy GBSS gene having an expected maximum of four alleles, revealed a seemingly implausible complexity of up to 15 alleles. More detailed analysis, using combinations of different PCR amplifications to increase robustness of the analysis revealed that the observed hyper diversity was likely due to chimera formation caused by priming with incompletely extended PCR products from previous cycles (Supplementary Fig. 8) as has been previously observed for simultaneous amplification of highly homologous templates^24^.

## Discussion

Most agronomic traits are influenced by multiple loci and obtaining individuals harboring the desired alleles at these loci are very unlikely. In hybrid breeding crops, such as most cereals, genetic diversity at important loci is frequently fixed in inbreds that are then combined to provide homogenous and vigorous offspring thus blocking further breeding improvement. Outbreeding crops, such as tetraploid potato, harbor numerous allelic variants at important loci and agronomic performance of single individuals, propagated as clones (seed potato), is the result of a complex balance of alleles at a multitude of loci, which is perturbed by further crossing. In both instances, crop development may be accelerated by targeted genetic mutations in already existing elite varieties^25^ using genome editing technology, such as CRISPR/Cas9. In outbreeding crops, the opportunity for altering specific traits in existing elite cultivars holds the promise of increasing performance of specific traits, without compromising good agronomic performance. Potato is tetraploid and elite germplasms are extremely genetically diverse with observed SNP frequency between two individuals of 1 per 29 bp^3^. Thus, genome editing is particularly attractive in potato where good agronomic performance is the result of balancing such overwhelming genetic diversity.

In the present study, we devised schemes for efficient editing and editing assessment of high ploidy complex genomes, here in potato, which are characterised by a high SNP prevalence between alleles but also between different cultivars.

We performed a survey for assessing the endogenous U6 promotor repertoire in potato and retrieved both published and hitherto unpublished potato U6 promotor sequences, and assessed their conferred editing performance in protoplasts as a first readout. Replacement of the regularly used Arabidopsis *AtU6*-*1* promotor with endogenous potato *StU6* promotors resulted in a dramatic increased editing efficiency of the target GBSS gene. Editing frequencies of 30–70% in protoplasts, corresponding to an up to a ca. 9 fold increased editing, were obtained as evidenced by IDAA. Theoretically 50% editing in protoplasts would translate into 6.25% (0.5^4^) ex-plants with full allelic editing. However, in agreement with earlier CRISPR/Cas editing in potato^13,14^, we observed full allelic editing in 35% of the ex-plants/shoots analysed, thus suggesting that in transformed protoplasts there is an increased likelihood that multiple alleles will undergo editing.

Our implementation of the IDAA technique permits fast and high throughput assessment of editing in organisms with high ploidy and complex genomes, such as potato, as evidenced here where all peak positions of the WT chromatogram were shifted in full allelic knock out of explants (Fig. 2). Editing may be scored at both the protoplast and ex-plant level, without the need for comprehensive sequence analysis, which, however, is still needed for full characterization at the ex-plant level.

Somaclonal variation has been reported to be a concern in relation to ex-plant regeneration from callus/shoots, including potato^26^. The present study did not include a comprehensive survey in regard hereto, but the regenerated first generation ex-plants displayed vigorous growth *in vitro* when scored ca 2 month after first visible shoot formation and later in pots in the greenhouse (Fig. 2A). Both the full allele GBSS edited ex-plants, such K10 and K33, and un-edited GBSS but regenerated plants, such as K34, for example, displayed the same vigorous growth under the growth and culturing conditions applied here. One ex-plant, K8 (depicted in insert to Fig. 2A), displayed a major growth phenotype, but further genotyping of this ex-plant was not pursued. Although somaclonal/phenotypic variation was generally not encountered in the present limited explant material, additional explant lines and propagation generations are needed for proper assessment of the extent of genetic as well as non-genetic derived somaclonal/penotypic variation.

In conclusion, this study demonstrates that *i)* use of endogenous *U6* promoters has an great impact on editing efficiencies, *ii)* in complex tetraploids an exhaustive molecular characterization of WT alleles is required to avoid SNP’s at gRNA targets and recombinant PCR derived erroneous allele assignment, *iii)* plasmid derived insertions were prevalent prompting for the use of purified DNA free nucleoprotein (RNP) to at least reduce the insertion prevalence, and *iv)* IDAA provides a simple qualitative and quantitative means of editing analysis at the cell pool and ex-plant level in the complex potato genome. The obtained highly improved editing efficacy may significantly reduce downstream cell culturing and ex-plant re-generation in particular where full allelic and heritable transmitted gene editing is desired.

## Methods

Methods and any associated references are available in the online version of the paper. Note: Any Supplementary Information and Source Data files are available in the online version of the paper.

### Online methods

#### Plant material

Sterile *in vitro* grown plantlets of Desirée and Wotan were obtained from Vitroform (Årslev, Denmark) and propagated in a Fitotron growth cabinet with 16/8 h, 24 °C/20 °C, 70% humidity, at 65μE light intensity as described in^12^. Regenerated plants were transferred to soil by rooting on peat under a white plastic cover. After rooting, plants were transferred to 25 cm pots and grown in the greenhouse to set tubers.

#### StU6 promoter sequence and cloning

*St*U6 promoter sequences were retrieved by BLAST searches in the reference genome (DM1–3 516 R44)^3^ with the sequences Z17290, Z17292, Z17293, Z17301 from the cultivar Record^18^ as baits. Z17290, Z17292, Z17293 and Z17301 were mapped to the reference genome using CLC Genomics Workbench (Qiagen). 5′ flanking sequences AGCAAGATGCAATGTATCAACTCA (Z17290), ACCACTTAAACTGAGAACAGTCAA (Z17292), TTCACTTAGTTCAGTTGCATTATGTC (Z17293), GATAAATTCTTAAAGTTGAGTAACC (Z17301) were used to amplify and sequence *U6* upstream regions from cultivars Desirée and Wotan of 421 bp (Z17290), 433 bp (Z17292), 372 bp (Z17293) and 374 bp, 321 bp and 318 bp (Z17301) using the common RV primer GCCATGCTAATCTTCTCTGTATCG that anneals to nt 33–55 of the *U6* sequence. These sequences were used to define *StU6*-*1* (Z17290), *StU6*-*2* (Z17292), *StU6*-*3* (Z17293) and *StU6*-*4*, *StU6*-*4a* and *StU6*-*4b* (Z17301), which were cloned to drive the gRNA expression using Nebuilder assisted Gibson assembly (Supplementary Fig. 2C).

#### In silico prediction analysis of GBSS-gRNA1 and 2 targets

Exon 1 of GBSS, which includes 36 potential gRNA targets, was used as input for evaluation of the ranking of gRNA1 and gRNA2. The *in silico* assisted gRNA selection tools^30^ amenable for plants and included in the survey were CHOPCHOP v2 (http://chopchop.cbu.uib.no/)^31^, CRISPR-P 2.0 (http://crispr.hzau.edu.cn/CRISPR2/)^32^, SSC (http://crispr.dfci.harvard.edu/SSC/)^33^, CRISPRater (https://crispr.cos.uni-heidelberg.de/)^34,35^, and CRISPOR (http://crispor.tefor.net/)^36^.

#### Protoplast isolation, transformation, editing, ex-plant regeneration

Protoplast isolation, transformation, editing and ex-plant regeneration were essentially done as described in^12^.

#### DNA extraction

Plant gDNA template for PCR and IDAA was purified with GenElute Plant genomic DNA miniprep kit from Sigma and quantified using nanodrop. gDNA from harvested protoplasts was obtained by suspending the cells in H_2_O, snap freezing in liquid nitrogen followed incubation at 96 °C for 15′.

#### Genotyping

To characterize the target region, different combinations of FW primers tgtagaccacacatcacATG, tgtagaccacacatcacATGG, GCAAGCATCACAGCTTCACACC, AGCATCACAGCTTCACACCACT and, + SNP FW, and RV primers atcatttagGCCCGCGGACA, AAACGTGGGGTTGATCGTGT, ATGGCCCCAAAGCTGGACTAG and + SNP RV, were used for PCR amplification with CloneAmp™ HiFi PCR Premix (Clontech) in total reaction volumes of 25 µl with 0.25 µM primers and 0.4–0.8 ng/µl gDNA according to the manufacturers recommendations with the PCR parameters of 35 cycles: 98 °C 10″, 64 °C 15″, 72 °C (60″ per 1000 bases, extension). In the attempts to reduce PCR derived chimeras, extension time was doubled and the primer concentration increased twofold. For genotyping of edited plants and protoplasts, the edited region was amplified with + SNP FW and + SNP RV. Gel purified PCR products were cloned using the CloneJet PCR cloning Kit (Thermo Scientific), sequenced by Sanger sequencing at Macrogen and analyzed on the CLC Main Workbench.

#### Screening by Indel Detection by Amplicon Analysis (IDAA)

Tri-primer PCR amplicons for IDAA^10^ were obtained using the primers + SNP FW, IDAA + SNP RV and Fluorescein Amidite (FAM) labelled FAMF, while primers –SNP FW and FAM-labelled -SNP RV were used for di-primer PCR. PCR reactions of 25 µl using CloneAmp™ HiFi PCR Premix (Clontech) with 0.25 µM primers except IDAA + SNP RV at 0.025 µM on a template of 0.4–0.8 ng/ µl gDNA or 2.5 µl protoplast cell pool suspension were run with PCR parameters: 98 °C 10″, 64 °C 15″, 72 °C (60″ per 1000 bases, extension) 35 cycles for gDNA, 40 cycles for protoplast suspensions. The PCR product was analysed on a 3500xL Genetic analyzer (Applied Biosystems) as previously described^10^.

#### Phenotypic analysis

Lugol staining was done as described in^29^.

## Supplementary information

Supplemental information
