# Supplementary material for: High efficacy full allelic CRISPR/Cas9 gene editing in tetraploid potato
Source: Sci Rep. 2019 Nov 27;9:17715. doi: 10.1038/s41598-019-54126-w (PMC6881354; doi:10.1038/s41598-019-54126-w)
Supplement: Supplementary file 1 — Supplemental information [file 41598_2019_54126_MOESM1_ESM.pdf]

Supplementary Information

High efficacy full allelic CRISPR/Cas9 gene editing in tetraploid potato

Ida Elisabeth Johansen[1], Ying Liu[1], Bodil Jørgensen[1], Eric Paul Bennett[2], Erik Andreasson[3], Kåre L. Nielsen[4], Andreas Blennow[1] and Bent Larsen Petersen[1]*

[1]Department of Plant and Environmental Sciences, University of Copenhagen, DK-1871 Frederiksberg C, Denmark

[2]Copenhagen Center for Glycomics, Department of Molecular and Cellular Medicine and School of Dentistry, Faculty of Health Sciences, University of Copenhagen, DK-2200 Copenhagen N, Denmark

[3]Resistance biology, Plant Protection Biology, Swedish University of Agricultural Sciences, Alnarp, Sweden

[4]Department of Chemistry and Bioscience, Aalborg University, Denmark

Correspondence should be addressed to Bent Larsen Petersen (blp@plen.ku.dk)

Table of Contents

Supplementary Fig. 1. *StU6 promoter nucleotide alignment*

Supplementary Fig. 2. *StU6 promoter cloning strategy*

Supplementary Fig. 3. *StU6-2-4 promoter mediated editing efficacies at the cell pool level*

Supplementary Fig. 4. *StU6-1-4 promoter efficacy test on second gRNA target*

Supplementary Fig. 5. *In silico prediction analysis of the two GBSS gRNA targets*

Supplementary Fig. 6. *Scheme for single cell transformation and clonal ex-plant propagation*

Supplementary Fig. 7. *Molecular and phenotypic analysis of residual edited ex-plants*

Supplementary Fig. 8. *Genotyping - PCR derived overestimation of allele complexity*

References

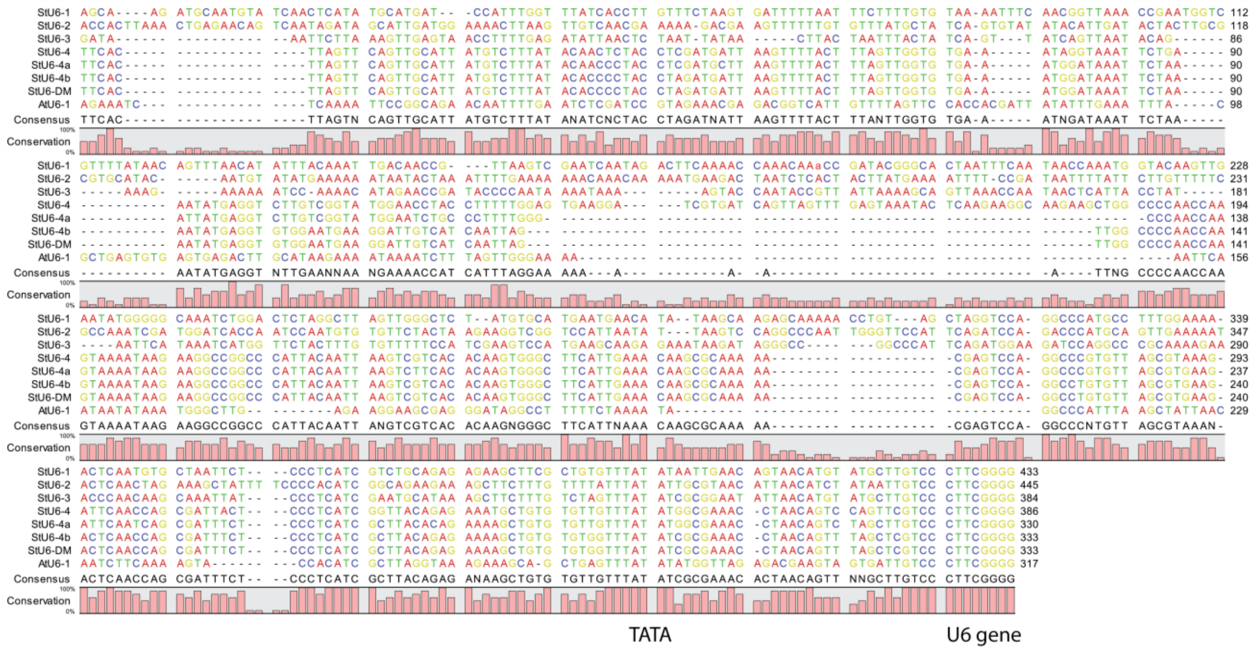

Supplementary Figure S1. *StU6 promoter nucleotide alignment*

Alignment of sequences upstream the *U6* gene transcription start (GTCCCTTCGGGG) from cultivar Wotan with the *StU6* DM (Phureja DM1-3 516 R44) promoter provided in[2] and *AtU6*-1.

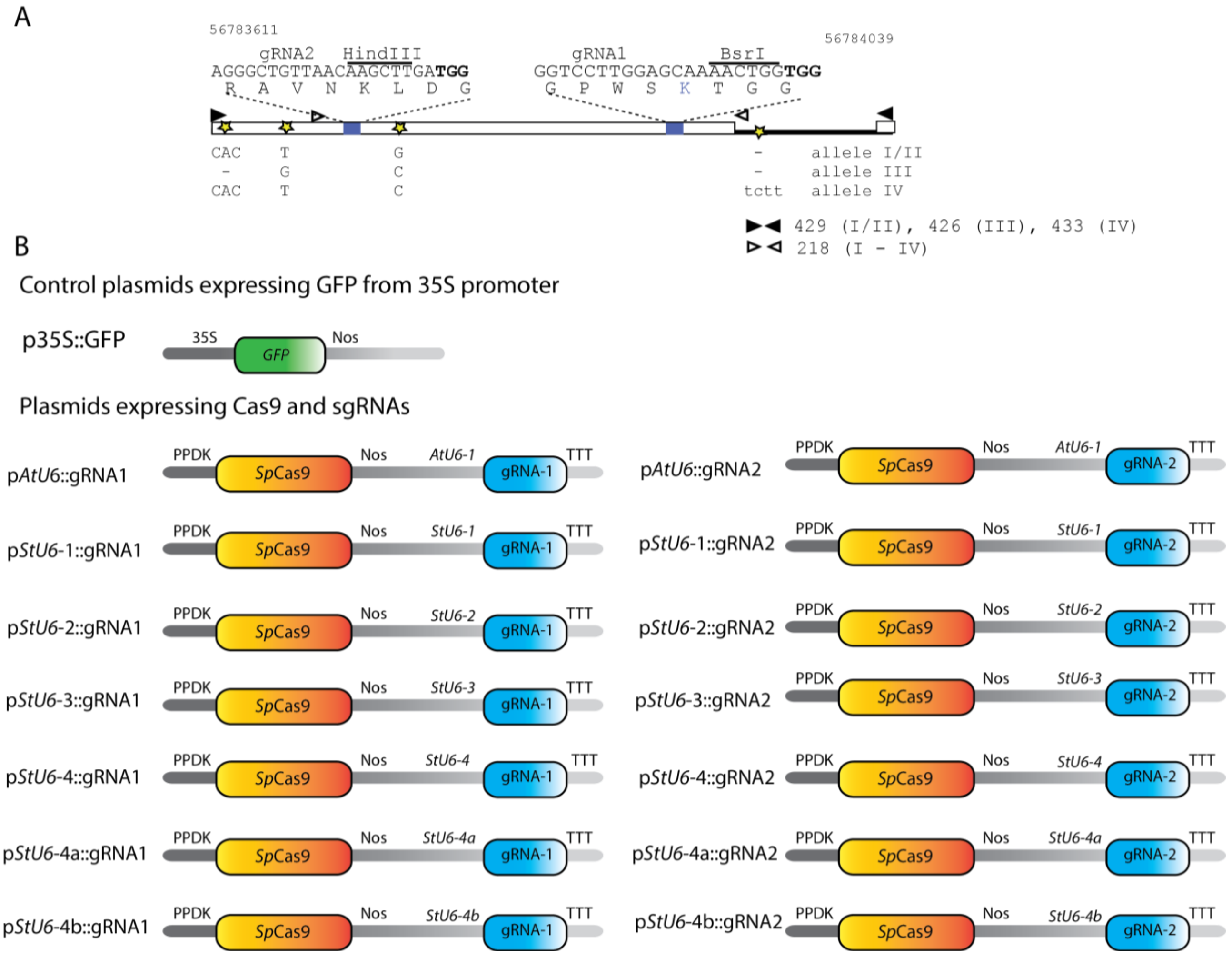

C

**Sequence of gRNAs**	
gRNA 1	5’- GGTCCTTGGAGCAAAACTGG-3’
gRNA 2	5’-GAGGGCTGTTAACAAGCTTGA-3
**Diagnostic primers + / - SNP**	
+ SNP FW	5’-GGCAAGCATCACAGCTTCACAC-3
+ SNP RV	5-CGATGTCCGCGGGCCTAAATG-3
IDAA + SNP RV	*5’-AGCTGACCGGCAGCAAAATTG*CGATGTCCGCGGGCCTAAATG-3’
FAMF	5’-6-FAM-AGCTGACCGGCAGCAAAATTG-3’
- SNP FW	5’-CTGTTAACAAGCTTGATGGGCTCC-3’
- SNP RV	5’-6-FAM-ACTTACTGCAAGGGCTGGTGG-3’

D

U6 promoter sequences

AtU6-1 sequence:
AGAAACGAGACGGTCATTGTTTTAGTTCCACCACGATTATATTTGAAATTTACGTGAGTGTGAGTGAGACTTGCATAAGAAAATAAAATCTTTAGTTGGGA
AAAAATTCAATAATATAAATGGGCTTGAGAAGGAAGCGAGGGATAGGCCTTTTTCTAAAATAGGCCCATTTAAGCTATTAACAATCTTCAAAAGTACCACA
GCGCTTAGGTAAAGAAAGCAGCTGAGTTTATATATGGTTAGAGACGAAGTAGTGATT

StU6-1 sequence retrieved with Z17290:
TTTCTTTTGTGTAAAATTTCAACGGTTAAACCGAATGGTCGTTTTATAACAGTTTAACATATTTACAAATTGACAACCGTTAAGTCGAATCAATAGACTTC
AAAACCAAACAAACCGATACGGGCACTAATTTCAATAACCAAATGGTACAAGTTGAATATGGGGGCAAATCTGGACTCTAGGCTTAGTTGGGCTCTATGTG
CATGAATGAACATATAAGCAAGAGCAAAAACCTGTAGCTAGGTCCAGGCCCATGCCTTTGGAAAAACTCAATGTGCTAATTCTCCCTCATCGTCTGCAGAG
AGAAGCTTCGCTGTGTTTATATAATTGAACAGTAACATGTATGCTT

StU6-2 sequence retrieved with Z17292:
ATGCTATCAGTGTATATACATTGATACTACTTGCGCGTGCATACAATGTATATGAAAAAATAATACTAAATTTTGAAAAAAACAAACAAAAATGAAGACTA
ATCTCACTACTTATGAAAATTTTCCGATAATTTTATTCTTGTTTTTCGCCAAATCGATGGATCACCAATCCAATGTGTGTTCTACTAAGAAGGTCGGTCCA
TTAATATTAAGTCCAGGCCCAATTGGGTTCCATTCAGATCCAGACCCATGCAGTTGAAAAATACTCAACTAGAAAGCTATTTTCCCCACATCGGCAGAAGA
AAGCTTCTTTGTTTTATTTATATTGCGTAACATTAACATCTATAATT

StU6-3 sequence retrieved with Z17293:
ACCTTTTGAGATATTAACTCTAATTATAACTTACTAATTTACTATCAGTTATCAGTTAATACAGAAAGAAAAAATCCAAAACATAGAACCGATACCCCAAT
AAAATAAAAGTACCAATACCGTTATTAAAAGCAGTTAAACCAATAACTCATTACCTATAATTCATAAATCATGGTTCTACTTTGTGTTTTTCCATCGAAGT
CCATGAAGCAAGAGAAATAAGATAGGGCCGGCCCATTCAGATGGAAGATCCAGGCCCGCAAAAGAAACCCAACAAGCAAATTATCCCTCATCGAATGCATA
AAGCTTCTTTGTCTAGTTTATATCGCGGAATATTAACATGTATGCTT

StU6-4 sequence retrieved with Z17301:
TTCACTTAGTTCAGTTGCATTATGTCTTTATACAACTCTACCTCGATGATTAAGTTTTACTTTAGTTGGTGTGAAATAGGTAAATTCTGAAATATGAGGTC
TTGTCGGTATGGAACCTACCTTTTTGGAGTGAAGGATCGTGATCAGTTAGTTTGAGTAAATACTCAAGAAGGCAAGAAGCTGGCCCCAACCAAGTAAAATA
AGAAGGCCGGCCCATTACAATTAAGTCGTCACACAAGTGGGCTTCATTGAAACAAGCGCAAAAACGAGTCCAGGCCCGTGTTAGCGTAAAGATTCAACCAG
CGATTACTCCCTCATCGGTTACAGAGAAATGCTGTGTGTTGTTTATATGGCGAAACCTAACAGTCCAGTTC

StU6-4a sequence retrieved with Z17301:
TTCACTTAGTTCAGTTGCATTATGTCTTTATACACCCCTACCTAGATGATTAAGTTTTACTTTAGTTGGTGTGAAATGGATAAATTCTAAAATATGAGGTG
TGGAATGAAGGATTGTCATCAATTAGTTGGCCCCAACCAAGTAAAATAAGAAGGCCGGCCCATTACAATTAAGTCGTCACACAAGTGGGCTTCATTGAAAC
AAGCGCAAAAACGAGTCCAGGCCTGTGTTAGCGTGAAGACTCAACCAGCGATTTCTCCCTCATCGCTTACAGAGAAAAGCTGTGTGTGGTTTATATCGCGA
AACCTAACAGTTTAGCTC

StU6-4b sequence retrieved with Z17301:
TTCACTTAGTTCAGTTGCATTATGTCTTTATACAACCCTACCTCGATGCTTAAGTTTTACTTTAGTTGGTGTGAAATGGATAAATTCTAAATTATGAGGTC
TTGTCGGTATGGAATCTGCCCTTTTGGGCCCAACCAAGTAAAATAAGAAGGCCGGCCCATTACAATTAAGTCTTCACACAAGTGGGCTTCATTGAAACAAG
CGCAAAAACGAGTCCAGGCCCGTGTTAGCGTGAAGATTCAATCAGCGATTTCTCCCTCATCGCTTACACAGAAAAGCTGTGTGTTGTTTATATGGCGAAAC
CTAACAGTCTAGCT

Supplementary Figure S2. Guide RNA targets, construct design and sequences.
A. Position and sequences of gRNA1 and gRNA2 in GBSS exon 1, +SNP primers (filled triangles), -SNP primers (open triangles) and SNP's (stars) in exon 1 and intron 1 of the four alleles. **B**. Constructs with different U6-promoter-gRNA were assembled in vector pHBT- pcoCas9[4] by NeBuilder assisted Gibson assembly using primers provided in C and promoter sequence information provided in **D**. **C**. Sequences of gRNAs, analytical PCR + SNP and – SNP primers, and **D**, *StU6* promoter sequences, which were used in plasmid constructs to drive gRNA expression.

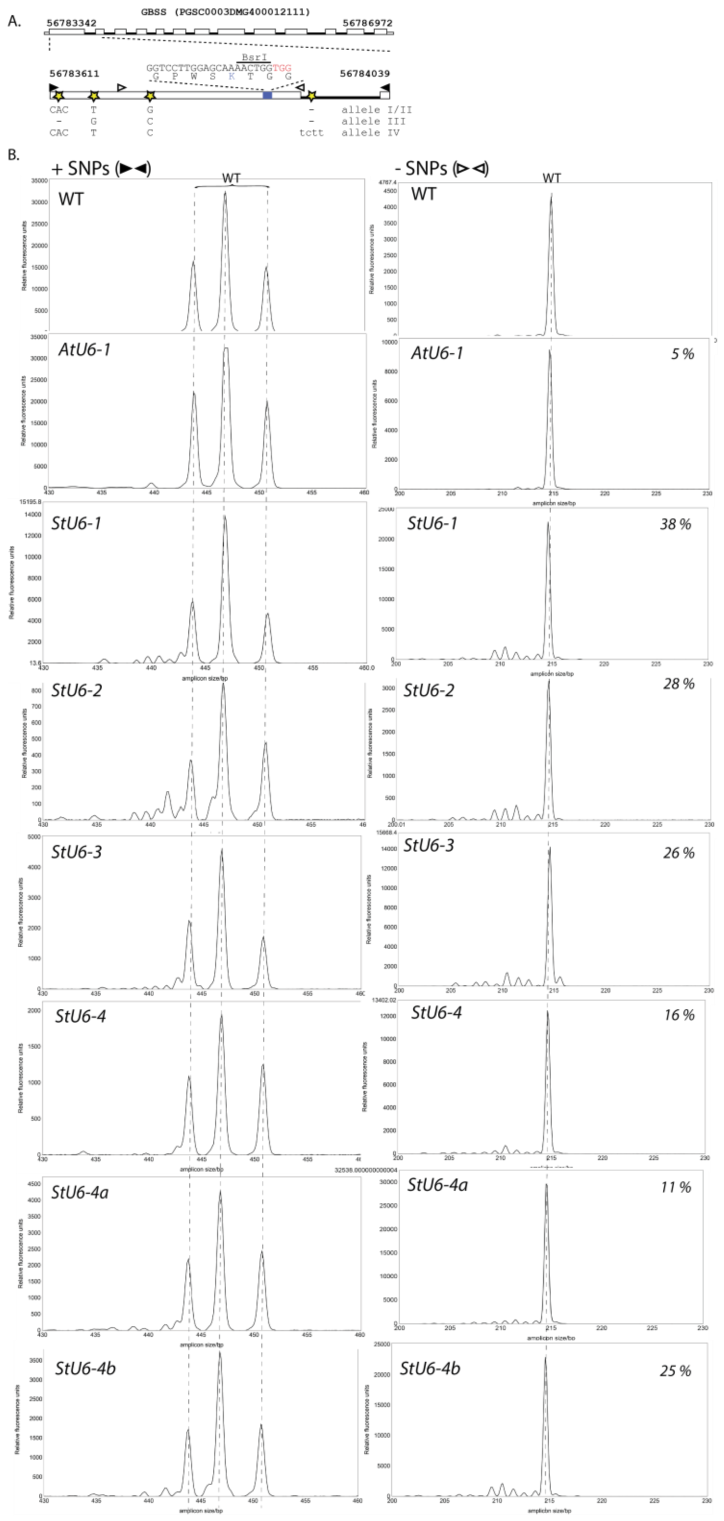

Supplementary Figure S3. Indel Detection by Amplicon Analysis (IDAA) at the cell pool level.

A. The GBSS gene and the edited region displaying gRNA1, + SNP primers (filled triangles) and – SNP primers (open triangles) used for IDAA PCR. **B**. IDAA on from *S. tuberosum* cv. Wotan protoplasts harvested 24 hours after PEG transformation with constructs expressing Cas9 and gRNA1 driven by *AtU6*-1 or *StU6* promoters. WT of unedited protoplasts were transformed with a plasmid expressing GFP. Amplification of the region including the length SNPs (+ SNPs, left) display allele complexity and inner (- SNP, right) allow quantification of editing as indel to WT ratios. WT peak positions are indicated by dotted lines. Indel formation in protoplasts of Wotan was generally lower than in those of Desiree.

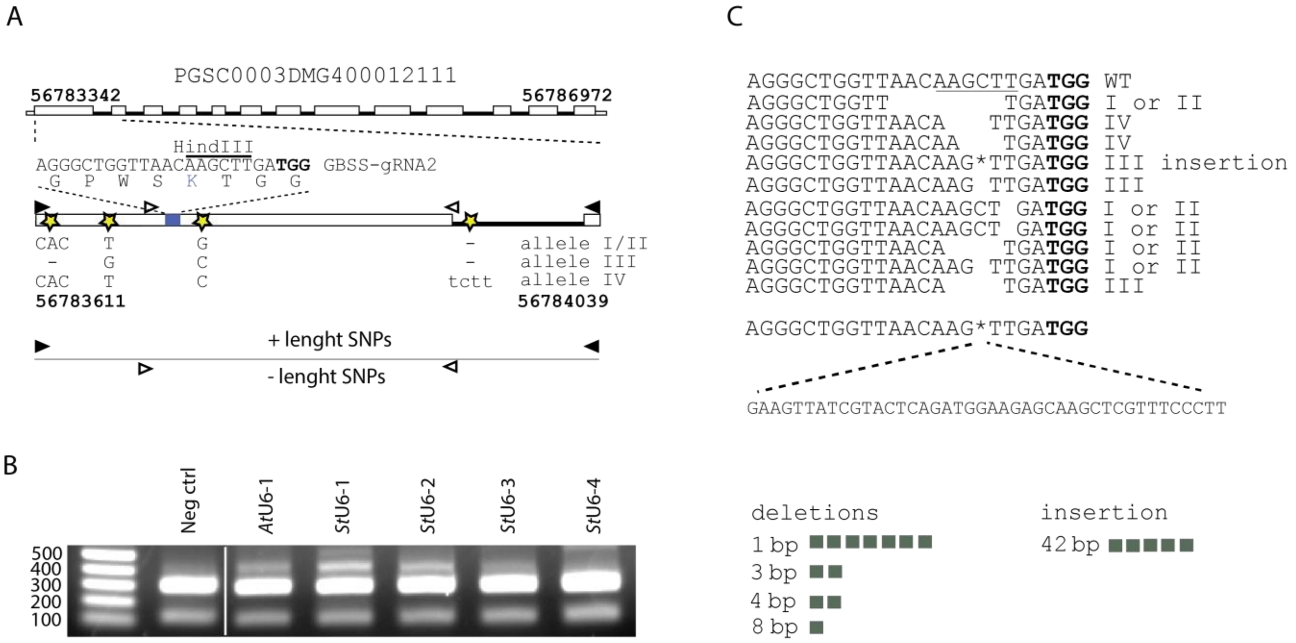

Supplementary Figure S4. *StU6-1-4 promoter efficacy test on second gRNA target at the cell pool level*

A. The GBSS gene and the edited region displaying gRNA2, +SNP primers (filled triangles), -SNP primers (open triangles). **B**. Efficacy test of GBSS-gRNA2 as evidenced by the presence of HindIII resistant bands (429 (I/II), 425 (III),, 432 (IV)). HindIII digestion of WT bands yields the fragments, 301, 128 (I/II), 301,124 (III), 305, 127 (IV). **C**. Sequence analysis of indels obtained from isolation and cloning of the *Hind*III resistant band of the *StU6*-2 experiment. Insertion position for the 42 bp insertion indicated by *.

Tool name	Model type	URL	Off target potato	gRNA1		gRNA2	
				Efficacy	Specificity	Efficacy	Specificity
SSC[5]	Learning	http://crispr.dfci.harvard.edu/SSC/	no	1		13	
CHOPCHOP v2[6]	Hypothesis	http://chopchop.cbu.uib.no/	no	1		10	
CRISPRater[7]	Hypothesis	https://crispr.cos.uni-heidelberg.de/	no	11		21	
CRIPSPR-P 2.0[8]	Alignment	http://crispr.hzau.edu.cn/CRISPR2/	yes		23		30
CRISPOR[9]	Alignment	http://crispor.tefor.net/	yes	1	24	19	12

>GBSS - exon 1 (NW_006238976.1)
ATGGCAAGCATCACAGCTTCACACCACTTTGTGTCAAGAAGCCAAACTTCACTAGACACCAAATCAACCTTGTCACAGATA
GGACTCAGGAACCATACTCTGACTCACAATGGTTTA**AGGGCTGTTAACAAGCTTGA**TGGGCTCCAATCAAGAACTAATACT
AAGGTAACACCCAAGATGGCATCCAGAACTGAGACCAAGAGACCTGGATGCTCAGCTACCATTGTTTGTGGAAAGGGAATG
AACTTGATCTTTGTGGGTACTGAGGTT**GGTCCTTGGAGCAAAACTGG**TGGACTAGGTGATGTTCTTGGTGGACTACCACCA
GCCCTTGCA

Supplementary Figure S5. In silico ranking of GBSS-gRNA1 and gRNA2.
In silico ranking of GBSS-gRNA1 and gRNA2 using SSC, CHOPCHOP v2 and CRISPRater that do not allow off-target screening in potato and CRISPR-P 2.0 and CRISPOR that include off-target screening against published potato genome sequence. While CRISPR-P 2.0 only provide a specificity score, CRISPOR give both a specificity score and an efficiency score for guide RNA expressed in cells.

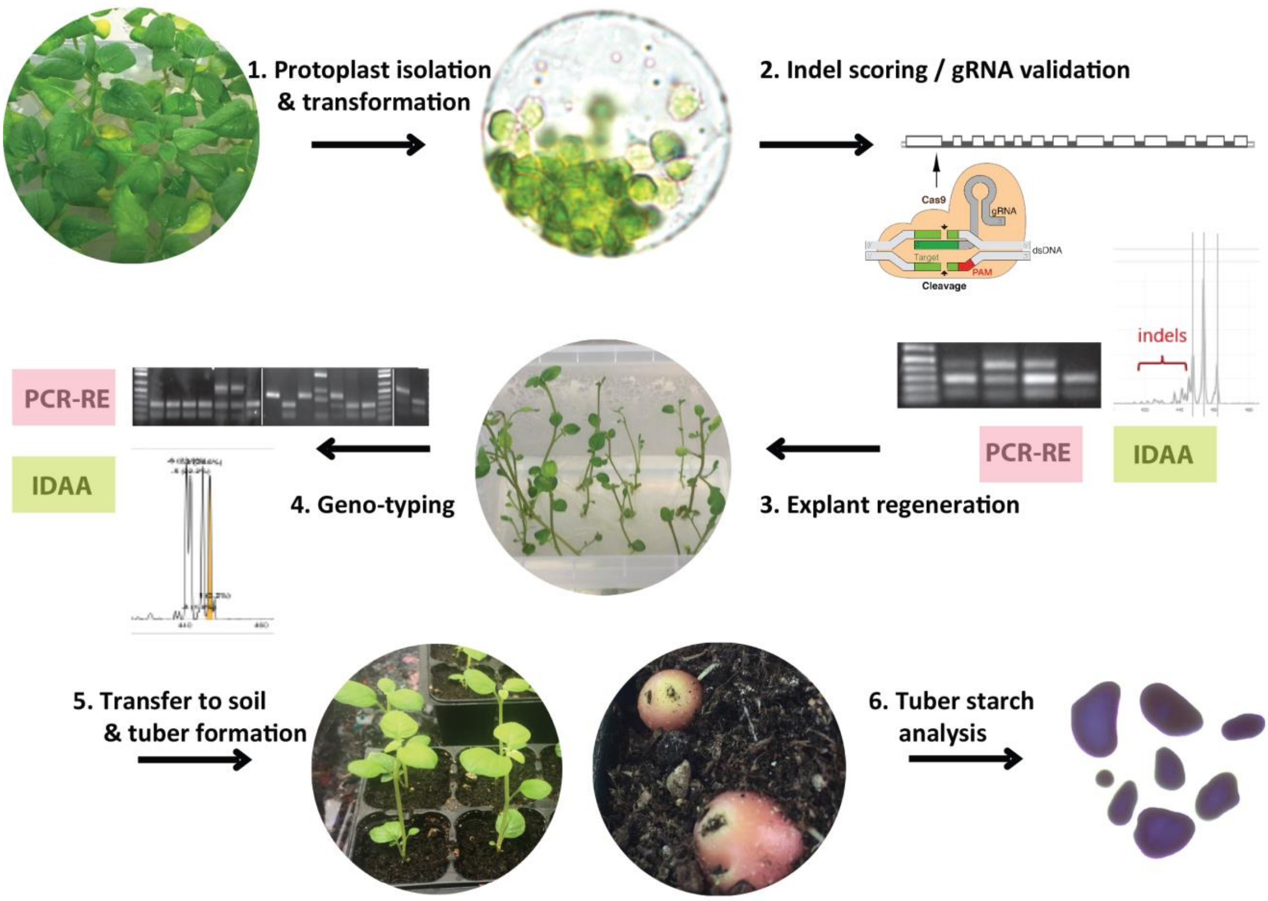

Supplementary Figure S6. Scheme for protoplast transformation, indel screening and explant propagation.
The scheme for single cell/protoplast transformation and following clonal ex-plant propagation may be divided into the following steps, 1. Protoplast generation and poly ethylene glycol (PEG) mediated transformation of CRISPR/Cas expression plasmids; 2. gRNA validation *in vivo* at the cell pool level (diagnostic IDAA and/or restriction enzyme analysis for indel scoring); 3. Explant regeneration from single protoplast through embedding in alginate and subsequent callus formation followed by shoot/explant induction; 4. Geno- and phenotyping of explants eliminating dwarfed plants and plants with insertions; 5 transfer to soil and tuber formation; 6. Starch analysis. Steps 1-2 are iterated until gRNA and transformation conditions with high editing efficacy are obtained, before entering procedures for clonal ex-plant selection and propagation.

Johansen et al,
Supplementary Figure 7

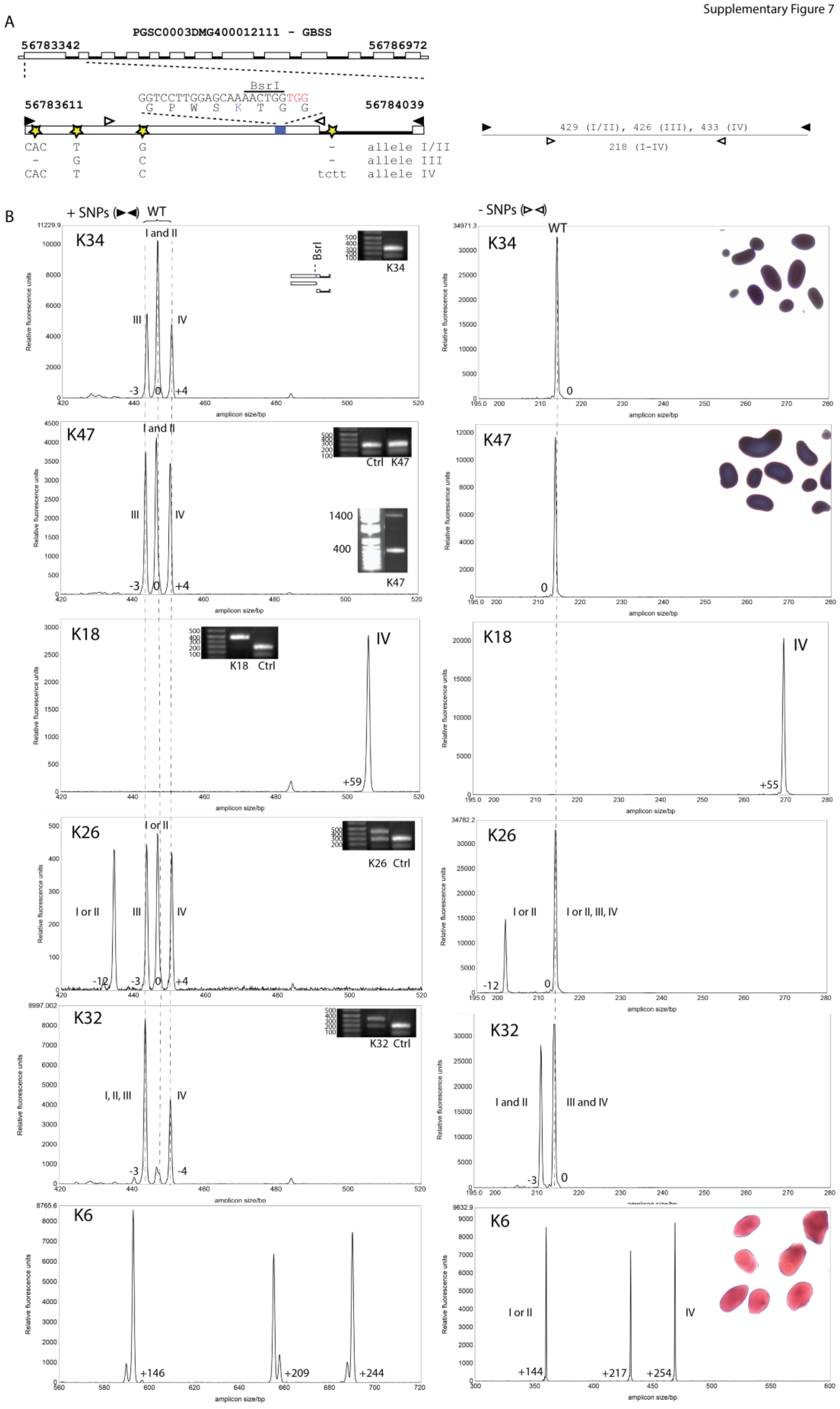

C.

	IDAA	Seq	Explant	Characterisation	Lugol
K6	+	12 seqs	+	8 sequences of I or II (144 bp insertion of Cas9 coding), 4 sequences of IV (254 bp insertion of plasmid U6 gRNA). III was not amplified	+
K10	+	+	+	3 nt deletion in III, I or II and IV; 990 bp insertion in I or II	+
K18	+	14 seqs	-	55 nt insertion in IV, III and I or II were not amplified, probably due to large insertions / deletions in the other three alleles. No WT alleles were encountered.	-
K26	+	-	-	12 nt deletion in I or II	-
K32	+	-	-	3 nt deletion in I or II	-
K33	+	+	+	6 nt deletion in I or II, 5 nt deletion in second I or II 4 nt deletion in IV, 1 nt insertion in III	+
K34	+	+	+	No editing	+
K47	+	+	+	> 3 kbp insertion of Cas9 coding sequence in I or II	+

Supplementary Figure S7. Molecular and phenotypic analysis of selected explants.
A. The GBSS gene and the edited region displaying gRNA1, +SNP primers (filled triangles) and –SNP primers (open triangles) used for IDAA PCR, **B**. IDAA and *Bsr*I restriction enzyme analysis (gel photo inserts) of *S. tuberosum* cv. Wotan ex-plants K6, K18, K26, K32, K34, and K47 and lugol stained starch grains from tubers of K6, K34 and K47 (inserts). Amplification of the region including the length SNPs (+SNPs, left) displays allele complexity and amplification of the inner (-SNP, right) region allows for more direct assessment of editing including indel to WT ratios. WT peak positions are indicated by dotted lines. **C**. Table, summarizing ex-plant data.

A

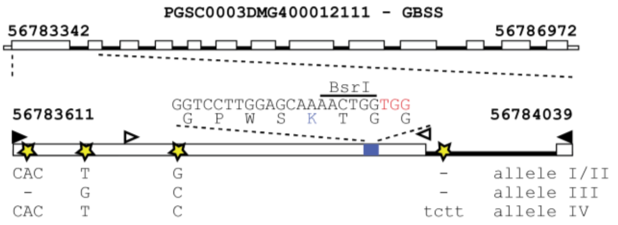

5678xxxx	3633-5	3722	3760	3948	4073	4092	4097	4111-2	4125	4129-31	4144	4148	4158	4174-82	4191	4228
TG0	CAC	T	G	-	C	A	A	at	a	gtt	c	a	a	ctctacttt	G	T
TC4	CAC	T	C	tctt	C	G	T	at	a	gtt	t	a	g	del	G	T
GC0	-	G	C	-	T	A	T	tc	g	a	t	c	a	ctctacttt	A	A
	CAC	T	G	-	T	A	T	tc	g	a	t	c	a	ctctacttt	A	A
	CAC	T	G	tctt	C	G	T	at	a	gtt	t	a	g	del	G	T
	CAC	T	G	-	C	A	A	at	a	gtt	t	a	g	del	G	T
	CAC	T	G	-	C	A	A	at	a	gtt	c	a	a	ctctacttt	G	A
	CAC	T	C	-	C	A	A	at	a	gtt	c	a	a	ctctacttt	G	T
	CAC	T	C	tctt	C	G	T	at	a	gtt	t	a	a	ctctacttt	G	T
	CAC	T	C	-	T	A	T	tc	g	a	t	c	a	ctctacttt	A	A
	CAC	T	C	tctt	T	A	T	tc	g	a	t	c	a	ctctacttt	A	A
	-	G	C	-	C	A	A	at	a	gtt	c	a	a	ctctacttt	G	T
	-	G	C	tctt	C	G	T	at	a	gtt	t	a	g	del	G	T
	CAC	G	C	-	T	A	T	at	a	gtt	c	a	a	ctctacttt	G	T
	CAC	G	G	-	C	A	A	at	a	gtt	c	a	a	ctctacttt	G	T

B

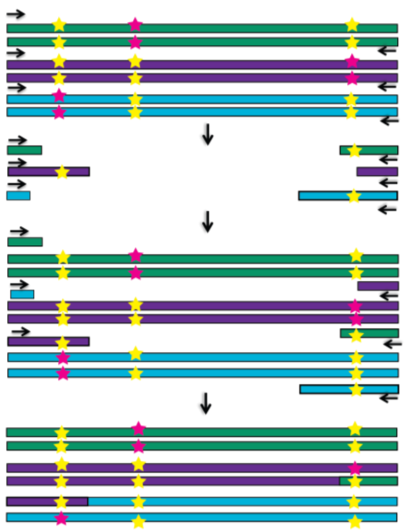

Supplementary Figure S8. Genotyping - PCR derived overestimation of allele complexity

Genotyping of the target region in GBSS by PCR amplification, cloning and sequencing suggested a higher complexity than could be explained by the presence of four alleles. **A**. Sequence characterization of the first four exons and three introns identified the three alleles I and II, IV and III, found in a ratio of 2:1:1 in a total of 80 sequences but also 12 chimeric variants representing 10-15% of the sequences but each only identified in one or two sequences (nucleotide polymorphisms, within the diagnostic PCR for editing analysis, including the gRNA1 target sequence, are shown. 'TG0', 'TC4' and 'GC0' at the left designate the overall nucleotide difference between the alleles and corresponds to the alleles I and II, IV and III, respectively. **B**. Illustration of how PCR amplification of almost identical templates may lead to chimera formation[10]. Attempts to reduce the problem by increasing primer concentration and annealing temperature and reducing amplification cycles were unsuccessful (see online methods). Deep sequencing or as applied here, a high number of PCR combinations and replicates, appeared the only viable way of obtaining a robust characterization of the target region.

References

1. Guerineau, F. & Waugh, R. The U6 small nuclear RNA gene family of potato. *Plant Mol Biol* **22**, 807-818 (1993).
2. Wang, S. et al. Efficient targeted mutagenesis in potato by the CRISPR/Cas9 system. *Plant Cell Rep* **34**, 1473-1476 (2015).
3. Andersson, M. et al. Efficient targeted multiallelic mutagenesis in tetraploid potato (Solanum tuberosum) by transient CRISPR-Cas9 expression in protoplasts. *Plant Cell Rep* **36**, 117-128 (2017).
4. Li, J.F. et al. Multiplex and homologous recombination-mediated genome editing in Arabidopsis and Nicotiana benthamiana using guide RNA and Cas9. *Nat Biotechnol* **31**, 688-691 (2013).
5. Xu, H. et al. Sequence determinants of improved CRISPR sgRNA design. *Genome Res* **25**, 1147-1157 (2015).
6. Labun, K., Montague, T.G., Gagnon, J.A., Thyme, S.B. & Valen, E. CHOPCHOP v2: a web tool for the next generation of CRISPR genome engineering. *Nucleic Acids Res* **44**, W272-276 (2016).
7. Labuhn, M. et al. Refined sgRNA efficacy prediction improves large- and small-scale CRISPR-Cas9 applications. *Nucleic Acids Res* **46**, 1375-1385 (2018).
8. Liu, H. et al. CRISPR-P 2.0: An Improved CRISPR-Cas9 Tool for Genome Editing in Plants. *Mol Plant* **10**, 530-532 (2017).
9. Prykhozhij, S.V., Rajan, V., Gaston, D. & Berman, J.N. CRISPR multitargeter: a web tool to find common and unique CRISPR single guide RNA targets in a set of similar sequences. *PLoS One* **10**, e0119372 (2015).
10. Smyth, R.P. et al. Reducing chimera formation during PCR amplification to ensure accurate genotyping. *Gene* **469**, 45-51 (2010).
